# Tumor Heterogeneity in Gastrointestinal Cancer Based on Multimodal Data Analysis

**DOI:** 10.3390/genes15091207

**Published:** 2024-09-13

**Authors:** Dongmei Ai, Yang Du, Hongyu Duan, Juan Qi, Yuduo Wang

**Affiliations:** 1 School of Mathematics and Physics, University of Science and Technology Beijing, Beijing 100083, China; m202310814@xs.ustb.edu.cn (Y.D.); m202210804@xs.ustb.edu.cn (J.Q.); s20190829@xs.ustb.edu.cn (Y.W.); 2 Department of Statistics and Financial Mathematics, School of Mathematics, South China University of Technology, Guangzhou 510641, China; 202310187412@mail.scut.edu.cn

**Keywords:** tumor heterogeneity, transcriptome profile, cancer classification, multiomics, cancer imaging

## Abstract

Background: Gastrointestinal cancer cells display both morphology and physiology diversity, thus posing a significant challenge for precise representation by a single data model. We conducted an in-depth study of gastrointestinal cancer heterogeneity by integrating and analyzing data from multiple modalities. Methods: We used a modified Canny algorithm to identify edges from tumor images, capturing intricate nonlinear interactions between pixels. These edge features were then combined with differentially expressed mRNA, miRNA, and immune cell data. Before data integration, we used the K-medoids algorithm to pre-cluster individual data types. The results of pre-clustering were used to construct the kernel matrix. Finally, we applied spectral clustering to the fusion matrix to identify different tumor subtypes. Furthermore, we identified hub genes linked to these subtypes and their biological roles through the application of Weighted Gene Co-expression Network Analysis (WGCNA) and Gene Ontology (GO) enrichment analysis. Results: Our investigation categorized patients into three distinct tumor subtypes and pinpointed hub genes associated with each. Genes *MAGI2-AS3*, *MALAT1*, and *SPARC* were identified as having a differential impact on the metastatic and invasive capabilities of cancer cells. Conclusion: By harnessing multimodal features, our study enhances the understanding of gastrointestinal tumor heterogeneity and identifies biomarkers for personalized medicine and targeted treatments.

## 1. Introduction

Colorectal cancer (CRC) and gastric cancer (STAD) are the foremost gastrointestinal malignancies, and gastrointestinal cancer has the highest incidence rate among all cancers [1]. Gastrointestinal cancers, including esophageal, stomach, colonic, and rectal malignancies, account for over a million deaths annually [2,3]. In China, CRC and STAD exhibit heightened incidence and mortality rates [4]. A pronounced reciprocal relationship has developed between CRC and STAD. CRC frequently emerges as a subsequent primary malignancy in STAD patients, and STAD is the most common initial cancer in CRC patients [5,6,7]. Moreover, CRC and STAD share numerous similarities, including pathogenesis, pathological features, treatment approaches, and cellular profiles [8,9]. Hence, a comprehensive analysis of samples from CRC and STAD patients would not only facilitate the discovery of common features but would also provide a basis for improving the relevance and efficacy of therapeutic strategies to treat cancer.

In 2013, Singaporean researchers were the first to classify gastric cancer based on genomic expression, identifying three primary molecular subtypes: proliferative, metabolic, and mesenchymal [10]. In the following year, The Cancer Genome Atlas (TCGA) research consortium expanded this classification by employing six distinct molecular biology techniques to categorize gastric cancer into four additional molecular subtypes: chromosomal instability (CIN), microsatellite instability (MSI), genomic stability (GS), and Epstein-Barr Virus positive (EBV+) [11]. Budinska et al. [12] reported five distinct subtypes of CRC by analyzing the expression profiles of 1113 colorectal cancer (CRC)-related genes. This expanded classification system presented significant variations in biological traits, clinical outcomes, pathological features, and survival data. CRC was further stratified into four Consensus Molecular Subtypes (CMS) at the molecular level, each distinguished by its oncogenic and oncostatic pathways, mutation profiles, microsatellite instability status, and clinical outcome expression patterns [13,14]. Utilizing miRNA data from colorectal cancer, Paz-Cabezas et al. [15] identified three distinct miRNA-driven tumor subtypes via hierarchical cluster analysis, which showed a strong correlation with mRNA-based tumor classifications (*p* < 0.001). These findings underscore the pivotal role of transcriptomic data in the identification of tumor subtype biomarkers.

Tumor imaging data are instrumental in revealing the spatial architecture, tissue composition, morphology, and internal organization of tumors, offering vital insights for cancer diagnosis. In the analysis of medical image, edge feature extraction stands out as an essential technique that aids in the identification and characterization of diseases. Several edge-detection algorithms are extensively utilized, such as the Sobel [16], Roberts [17], Prewitt [18], and Canny [19] operators. Histopathological images are pivotal in cancer classification and subtyping, enabling a more nuanced understanding of cancer heterogeneity [20]. For instance, N. K. Pratiwi and colleagues utilized the Canny operator to extract edge features from colon cancer images and subsequently applied these features to a classification study of colon cancer [21], thereby validating the efficacy of edge feature extraction in cancer diagnostics.

Multimodal data fusion effectively complements and integrates insights from different fields, including pathology, clinical radiology, genetics, and molecular biology [22], resulting in a more advanced and comprehensive analysis of heterogeneity in gastrointestinal cancers. However, integration of multi-omics datasets is challenging. In the present work, we addressed this complexity with an optimized Canny operator to increase the accuracy of extracting image information from tumor tissue sections. Subsequently, we integrated the extracted differential transcriptomes associated with gastrointestinal cancers, immune cell data, and corresponding tumor tissue images. By integrating these four types of data, we identified three distinct subtypes, each linked to specific hub genes that serve as key biomarkers for advancing personalized treatment. This analysis has deepened our understanding of the intrinsic heterogeneity within gastrointestinal (GI) tumors.

## 2. Materials and Methods

### 2.1. Datasets

In the present study, we used The Cancer Genome Atlas (TCGA) [23] to download mRNA and miRNA expression profiling data taken from primary tumor tissues of gastric and colorectal cancer patients. The mRNA and miRNA expression data were aligned to the human reference genome GRCh38. The mRNA dataset encompassed 937 samples, comprising 74 samples serving as normal controls, whereas the miRNA dataset comprised 964 samples with 53 normal controls. These samples corresponded to 58,387 mRNA transcripts and 2652 mature miRNAs. Furthermore, leveraging the LM22 gene set from the CIBERSORT algorithm, we estimated the relative abundance of 22 distinct immune cell types within the samples based on their mRNA expression profiles.

Imaging data were sourced from the research conducted by Kather et al. [24] and were retrieved from the publicly accessible Zenodo database at https://zenodo.org/records/2530789 (accessed on 10 August 2023). 

In sequence, our analysis of differentially expressed mRNAs, miRNAs, and immune cells used data from all samples. We specifically focused on gastrointestinal cancer patients, and we retained the sample at the intersection of the four data types for a total of 515 data samples for in-depth analysis.

### 2.2. Data Feature Extraction

#### 2.2.1. Edge Feature Extraction of Tumor Images

- Smooth images using bilateral filters

In 1986, John F. Canny introduced an algorithm, which was known as the Canny edge detection operator [25], for image edge detection. In the present work, we optimized this operator by replacing the conventional Gaussian filter, which was originally employed for preliminary filtering, with a more refined bilateral filter.

Bilateral filtering incorporates spatial information about pixel distribution, thus extending beyond the capabilities of the original Gaussian filter. This sophisticated method optimally refined edge and grayscale details, effectively reduced texture noise, and maintained crucial representative information, thereby efficiently suppressing noise [26]. The bilateral filtering formulas can be found in the Supplementary Materials.

Utilizing the “opencv” library, we loaded each sample image into a 224 × 224-pixel matrix, configuring the parameters to N=2, $\sigma_{d}=1,$ and $\sigma_{r}=1$. The origin of the pixel coordinate system was set at the lower-left corner and denoted as (0,0). Following this, we applied weighted averaging to process the pixel matrices of the images.

- Calculation of gradient change and direction of grayscale values

We applied the Sobel operator to compute the variations and orientations of gray-level values. The operator was utilized to determine the gradients across both positive and negative vertical axes on the horizontal plane. This process enabled us to ascertain the direction angle θ for each pixel.

Upon calculating the image gradients, it was possible that multiple directions could satisfy the threshold conditions. However, we selectively retained the gradient direction with the highest magnitude, while suppressing the others. The magnitude of the gradient at the current pixel was compared with the magnitudes in the four principal directions: 0 (vertical), $\frac{\pi }{4}$ (one of the diagonals), $\frac{\pi }{2}$ (horizontal), and $-\frac{\pi }{4}$ (the other diagonal). These are common edge directions in images. A pixel was deemed significant and retained if its gradient intensity was greater than the gradient intensities of the adjacent pixels in all four cardinal directions; if not, the pixel was discarded.

- Setting dual thresholds for edge detection

To refine the noise-reduction process, we introduced dual thresholds: a high threshold (TH) and a low threshold (TL). Edge pixels with gradient magnitudes exceeding TH were designated as strong edges, while those with magnitudes between TH and TL were categorized as weak edges. Pixels not meeting these criteria were effectively suppressed. The determination of TH and TL is outlined in the Supplementary Materials.

Differentiating weak edge pixels that belong to true edges from those caused by noise is essential for accurate edge detection. To this end, we set a criterion specifying that a weak edge pixel should be retained and considered part of the image’s edge structure, but only if it was connected to at least one pixel previously identified as a strong edge pixel.

Upon completing the aforementioned image-processing steps, we derived the final matrix representing the edge features of the image. We then computed the average value across each column of this matrix to achieve dimensionality reduction for the data corresponding to each sample. This process led to a dimensionally reduced size of 1 × 224 for the data of each sample.

In this study, “edge features” refer to the distinct boundaries and contours extracted from tumor images using the optimized Canny edge-detection algorithm. This is different from “edges” in network analysis, which refers to a relationship or connection between nodes. These features capture the spatial organization and morphology of the tumor, providing valuable insights into its structure and composition. Integrating image-based features with transcriptomic and immune cell data provides a multimodal view of gastrointestinal tumor heterogeneity.

#### 2.2.2. Transcriptome Feature Extraction

Given the multitude and complexity of mRNA and miRNA data, the presence of redundant or irrelevant features can potentially distort analytic outcomes. To address this, we opted for a feature dimensionality reduction strategy aimed at boosting the precision and efficiency of our research designed to explore the heterogeneity of GI cancer. Furthermore, we prioritized features that were significantly differentially expressed between cancer patients and healthy individuals since we hypothesized that these features might play a crucial role in disease diagnosis and the development of treatment strategies.

To discern variations in gene expression between cancerous and normal samples, we first conducted a differential analysis for mRNAs and miRNAs. This analysis was performed utilizing the limma package (version 3.58.1; R package from CRAN), employing both logFC (log2 fold change) and Bayesian statistical testing approaches [27,28]. More specifically, a gene was flagged as differentially expressed if it exhibited a logFC greater than 1 and yielded a *p*-value below 0.01 from the Bayesian test.

Through our analysis, we had identified 3360 downregulated and 2484 upregulated genes, totalling 5844 differentially expressed mRNAs. Additionally, we found 91 downregulated and 72 upregulated miRNAs, totalling 163 differentially expressed miRNAs. As presented in Figure S1, the heat maps display differential expression profiles based on samples from cancer patients and normal subjects, and, as such, validate the reliability of our differential characterization approach.

### 2.3. Multimodal Data Clustering

#### 2.3.1. Soft Threshold Distance Calculation

Soft threshold distance calculations were conducted autonomously for mRNA, miRNA, immune cell, and image datasets. We denoted n samples and m features, such as mRNA gene expressions, by matrix $Q_{m\times n}$, which served as the sample-feature matrix. We then computed the Pearson correlation coefficient matrices $A_{m\times m}={\left[a_{ij}\right]}_{m\times m}$ for each of the omics datasets independently.

Within the $S_{m\times m}$ matrix, elements underwent a nonlinear mapping process, which facilitated the incorporation of a soft threshold.

To ascertain the soft threshold, we utilized an approach akin to a grid search, delineating a spectrum of β values ranging from 2 to 20. The criteria for selecting the soft threshold were based on the condition that the coefficient of determination ($R^{2}$) for the linear regression model should exceed 0.8. In the absence of a satisfactory soft threshold, one was selected that corresponded to an average connectivity of fewer than 100 samples.

Soft thresholding was applied across four distinct data categories, yielding the following outcomes: a threshold of 8 for the mRNA data correlation coefficient matrix, 14 for the miRNA data correlation coefficient matrix, 3 for the image data similarity matrix, and 9 for the immune cell data.

#### 2.3.2. Calculation of DissTOM Distance for the Soft Threshold Matrix

We employed the Topological Overlap Matrix (TOM) [29,30,31] to delineate correlations among samples within the sample network. Thereafter, in the relational equation, we converted the adjacency matrix into the TOM matrix $W_{m\times m}={\left[w_{ij}\right]}_{m\times m}$ to more precisely capture the complex intersample relationships.

A direct relationship was indicative of exclusive connectivity or related pathways that existed between samples i and j when $w_{ij}=1$. Conversely, $w_{ij}=0$ signified the nonexistence of a relationship. Subsequently, we defined the dissimilarity measure $d_{ij}=1-w_{ij}$ to construct the dissimilarity matrix, dissTOM.

We employed the k-medoids algorithm to cluster dissTOM and utilized the elbow method to identify the optimal number of clusters for each class. To prevent an excessively high number of classes, we limited the range of *k* to between 2 and 10. The chosen value of k represented the point of maximum deviation in the sum of squared errors (SSE) for each data type. The clustering analysis concluded with *k* set to 5 for mRNA data, 4 for miRNA data, 4 for image data, and 3 for immune cell data.

#### 2.3.3. Construction of Similarity and Kernel Matrices

Inspired by the similarity network fusion (SNF) model used by Bo Wang et al. [32] in 2014, we calculated the similarity matrix $P_{m\times m}={\left[P_{ij}\right]}_{m\times m}$ by calculating the distance matrix $Q_{m\times m}={\left[q_{ij}\right]}_{m\times m}$ based on the dissTOM matrix. The distances between samples are transformed using a scaled exponential similarity kernel function, a modification of the Gaussian kernel. This transformation subtly transforms the data into a specific distribution within a smooth convex space, effectively describing the information more centrally [32]. Utilizing the preclustered data, we formulated the kernel matrix $S_{m\times m}={\left[S_{ij}\right]}_{m\times m}$. 

We iteratively refined $P_{v}$ for each data modality v = {mRNA, miRNA, image data, immune cell data} and quantified the iterative changes in the data in terms of Frobenius norms. The average of the four data modalities completed by the iteration was used as the fusion matrix. Formulas for the multimodal data fusion process can be found in the Supplementary Materials.

## 3. Results

### 3.1. Identification of the Three Subtypes Based on Sample Omics Data

**Figure 1:**
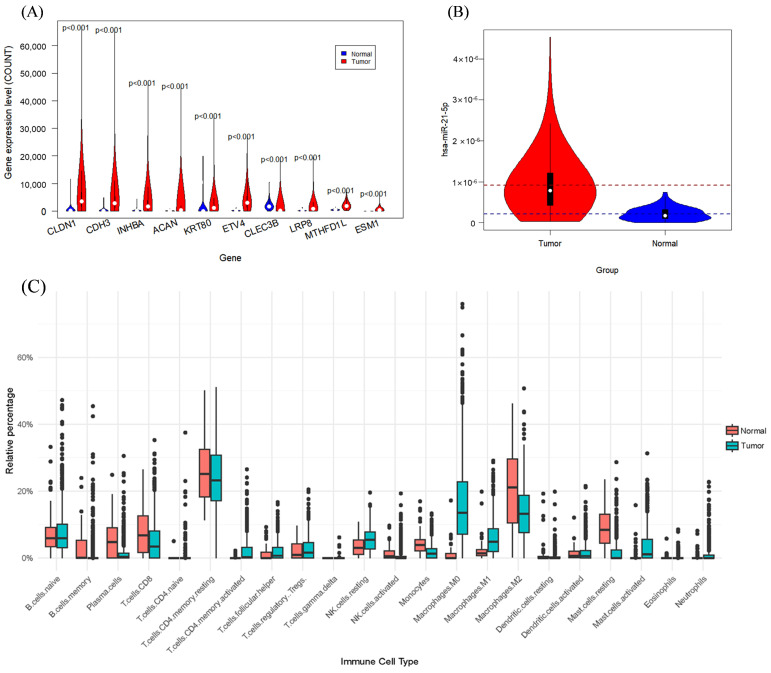
Significantly different characteristics between cancer and normal samples: (**A**) top 10 most significant mRNAs; (**B**) most significant differential miRNAs; (**C**) differential boxplots of 22 immune cells.

The violin plots depicting the top 10 most significantly differentially expressed mRNAs are presented in Figure 1A. Table S1 provides information on the top 20 most significant mRNAs. It is of particular interest that elevated expression levels of *CLDN3* in gastric cancer influence tumor cell permeability, facilitating their traversal across the basement membrane and extracellular matrix, thus potentially contributing to oncogenesis [33,34]. *CDH3*, a gene predominantly overexpressed in gastric cancer, is associated with cancer invasion and metastasis. The protein it encodes facilitates the proliferation and mobility of cancer cells [35].

Figure 1B illustrates the general upregulation of hsa-miR-21-5p, a microRNA (miRNA) that is expressed in a variety of cancers, especially in colorectal cancer, in which hsa-miR-21-5p contributes to tumor development and progression of tumors through the modulation of multiple biological processes, such as apoptosis and inflammatory responses [36]. Furthermore, we investigated the potential targets of miRNAs that are differently expressed. Notably, hsa-miR-21-5p, which is upregulated in colorectal cancer, has been shown to target genes involved in apoptosis and inflammatory responses, such as *TGFBI* and *PDCD4* [37,38], both of which were also found to be downregulated in our study. Table S2 lists information about the top 20 most prominent miRNAs.

Box plots of immune cell percentages for the two samples showed considerable variation in the proportion of certain immune cells, leading to the selection of data from 12 specific immune cells for further analysis.

Following four fusion iterations of the four data models, convergence was successfully attained. The iterative process, as depicted in Figure S2, illustrates the progressive convergence of the four similarity matrices across iterations. Subsequently, spectral clustering was employed to confine the class count (k) within the range of [2, 5], utilizing the elbow method to ascertain optimal k. In total, the study classified 515 samples into three subtypes, comprising 212, 132, and 171 samples, respectively. To more vividly exhibit the characteristic differences among the subtypes, the top 20 differential features of mRNA and RNA, along with the comprehensive data of immune cells, were filtered using the Kruskal–Wallis test.

**Figure 2:**
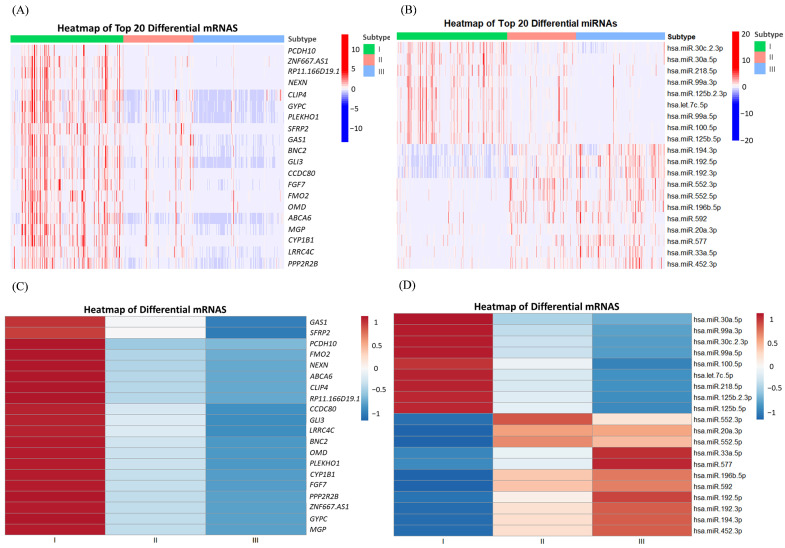
Heat maps of the top 20 significantly differentially expressed genes (DEGs): (**A**) mRNA expression heat map; (**B**) miRNA expression heat map; (**C**) mRNA expression heat map after taking the mean for the corresponding feature of the same subtype sample; (**D**) miRNA expression heat map after taking the mean for the corresponding feature of the same subtype sample.

**Figure 3:**
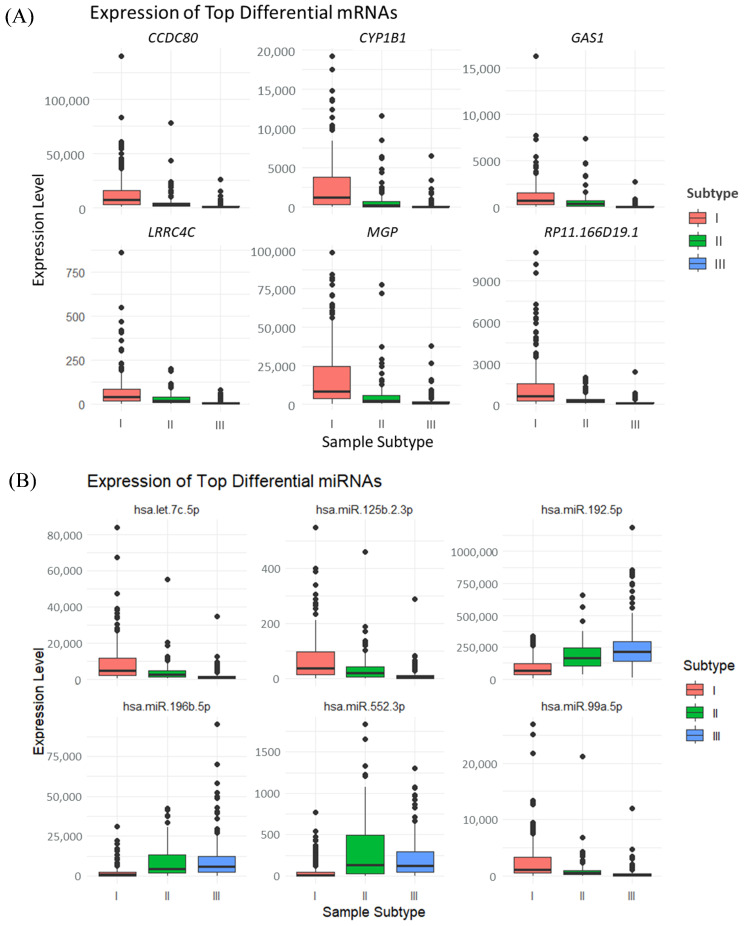
Six features with the most significant differences among the three subtypes: (**A**) Top six most significantly differentially expressed mRNAs; (**B**) Top six most significantly differentially expressed miRNAs.

Figure 2A,C illustrates the expression profiles of samples across different subtypes under the top 20 DEGS, with (C) presenting the mean expression values for samples within each subtype. In these mRNA expressions, samples from subtype I exhibit relatively high levels, while samples from subtype II show comparatively lower levels, and samples from subtype III have the lowest expression. Figure 2B,D depicts the expression of the first 20 differential miRNAs among samples of various subtypes, with (D) also presenting the average expression values for samples of each subtype. For the initial nine miRNA features, the expression levels across the three subtypes are presented in descending order, in alignment with the mRNA results. However, for the last 11 miRNA features, the expression outcomes are nearly inversely related. Corresponding box plots for the first six features are provided in Figure 3, revealing significant differences among subtypes, thereby preliminarily confirming the validity of the cancer-typing methodology followed in this study.

**Figure 4:**
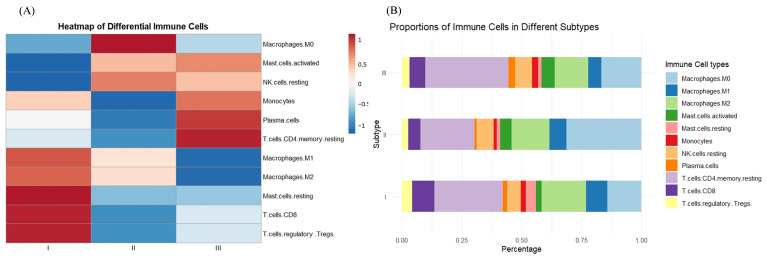
Immune cell characteristics of different subtypes: (**A**) Heat map of immune cell characteristics of samples of the same subtype after taking the mean value; (**B**) Difference in the percentage of immune cells in samples of different subtypes.

Figure 4 illustrates the expression patterns of various subtypes of immune cells, focusing on the first 11 classes identified through *p*-value testing. In subtype I, specific immune cells such as resting NK cells and plasma cells exhibit reduced activity (blue), whereas M2 macrophages and regulatory T cells (Tregs) display heightened activity (red). Subtype II presents a pattern distinct from that of subtype I, and subtype III diverges from subtype I in the activity of most cell types, with M1 and M2 macrophages demonstrating moderate to high activity, and resting NK cells and Tregs showing lower activity. As depicted in Figure 4B, subtype I is characterized by a higher proportion of M2 macrophages and Tregs, which may indicate a more potent anti-inflammatory or immunomodulatory role. Subtype II is marked by an increased presence of CD8 T cells and plasma cells, potentially linked to a more robust immune response or antibody production. In contrast, subtype III exhibits a higher proportion of M1 and M2 macrophages, which could be associated with tissue repair and modulation of the tumor microenvironment.

### 3.2. Identification of Hub Genes of Different Subtypes by WGCNA

**Figure 5:**
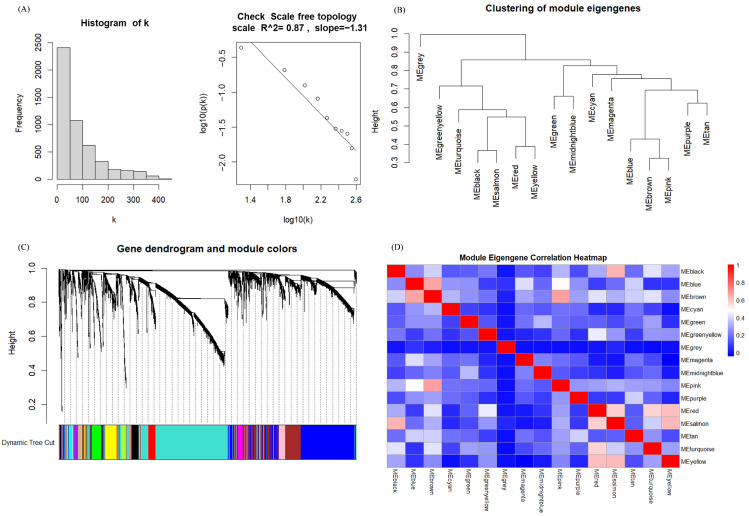
Results of Weighted Gene Co-expression Network Analysis (WGCNA): (**A**) Scale-Free Topology Analysis, frequency distribution of the number of connections (i.e., node degree, k) in the network (**left**), and a test of the scale-independent nature of the network (**right**); (**B**) Clustering of Module Eigengenes; (**C**) Gene Dendrogram and Module Colors, different colors represent different modules; (**D**) Module Eigengene Correlation Heatmap.

Weighted Gene Co-expression Network Analysis (WGCNA) was conducted on mRNA data based on the samples from three subtypes, encompassing a total of 515 samples and 5844 genes. Initially, during the analysis, 5000 genes with the highest variability were selected. Using the histogram algorithm, the soft-thresholding power β = 3 was identified, thereby achieving an R^2^ value of 0.88 and an average connectivity below 100 to meet our criteria (Figure S3). It can be observed in Figure 5A that (1) most mRNAs exhibit low connectivity, (2) only a minority demonstrates high connectivity, and (3) the constructed network exhibits scale-free properties. The dissTOM matrix was constructed by leveraging the topological overlap matrix (TOM) similarities to quantify gene-expression dissimilarities. This matrix forms the foundation for clustering and subsequent module identification, and the “cutreeDynamic” algorithm from the WGCNA package (version 1.72.5; R package from CRAN) was employed for dynamic pruning to discern 16 modules encompassing all genes. Module sizes ranged from a minimum of 33 genes to a maximum of 1747 genes with only 25 genes included in the gray module. The gray module was excluded from subsequent analyses, and the number of genes in each module is detailed in Table S3.

Utilizing the characteristics of Module Eigengenes (MEs), the correlation between individual genes and their corresponding modules can be precisely quantified. This correlation coefficient serves as a pivotal metric for assessing whether a gene functions as a hub gene within its module.

**Figure 6:**
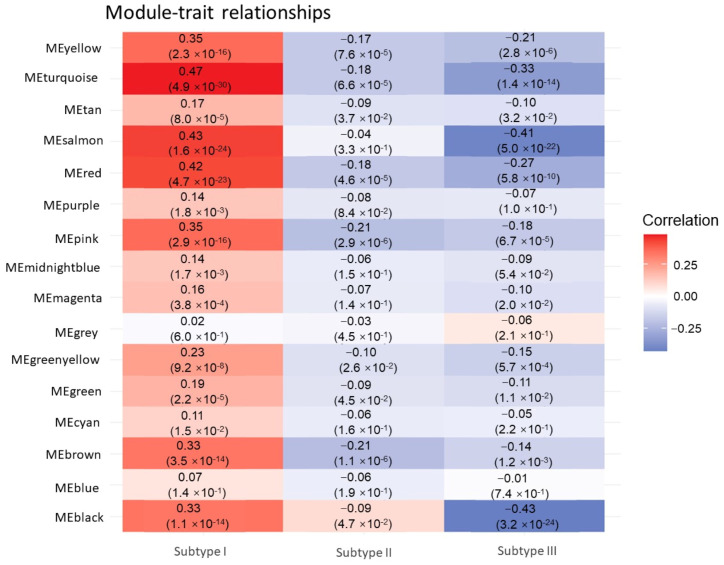
Correlation of different modules with different subtypes.

Figure 5B presents the correlation clustering tree dendrograms for the 15 identified modules, indicating a similarity threshold below 0.7 (with merge heights exceeding 0.3), thereby avoiding dynamic pruning. To pinpoint the key modules with the most robust correlations to sample traits, notably tumor subtypes, we assessed the gene module-sample subtype correlations (Figure 6). In this evaluation, categorical labels were assigned a value of 1, with non-relevant categories receiving 0. Employing a distinctive heat-coding strategy for labels, we conducted three separate analyses to determine the most pertinent central genes for each category. Pearson correlation coefficients were employed to gauge the relationship between feature genes, i.e., those linked to sample characteristics and the categorical variables denoting tumor subtypes. This methodology resulted in quantifying the correlation between tumor subtypes and feature genes across modules. The “Turquoise”, “Brown”, and “Black” modules demonstrated the most pronounced correlation with the three tumor subtypes. Within these pivotal modules, we initially determined the intramodular connectivity (kWithin) for each gene, reflecting the strength of its interaction with other genes within the same module, as calculated using the following formula:

(1) $$k_{i}=\sum_{j\in M}\left|c_{ij}\right|$$

where, $c_{ij}=corr(x_{i},x_{j})$ is the Pearson correlation coefficient within the module for the two genes.

The greater a gene’s intramodular connectivity, the more pivotal its role within the module and the more closely it interacts with other genes. We assessed each gene’s correlation with tumor subtypes (GS) and its agreement with the module’s signature genes (MM). Hub genes are characterized by high GS, high MM, and high within. Accordingly, for subtype I samples, genes were selected with GS > 0.45 and |MM| > 0.8; for subtype II samples, with GS > 0.2 and |MM| > 0.7; and for subtype III samples, with GS > 0.4 and |MM| > 0.8. Thereafter, by ranking genes based on kWithin in descending order, the study identified 16 hub genes for Subtype I, nine for Subtype II, and eight for Subtype III.

Table 1 presents an exhaustive compilation of hub genes for the three sample subtypes. Among the hub genes of subtype I, *MAGI2-AS3* facilitates the progression of gastric cancer by sequestering miR-141/200a, thereby sustaining the overexpression of *ZEB1* [39], an epitranscription factor that plays a role in the regulation of epithelial-mesenchymal transition (EMT), a pivotal process in cancer metastasis and invasion. *MAGI2-AS3*, through its interaction with miRNAs, is implicated in the modulation of the tumor microenvironment, impacting tumor cell proliferation, migration, and invasion. Concurrently, *MAGI2-AS3* advances the progression of colorectal cancer by manipulating the miR-3163/*TMEM106B* axis. It functions as a molecular sponge for miR-3163, inhibiting the suppressive effect of miR-3163 on *TMEM106B*, which results in the upregulation of *TMEM106B* expression and consequently fuels tumor cell proliferation and migration [40].

Among the hub genes of subtype II, *MALAT1* has been identified as intimately linked to the development, progression, and metastasis of various human cancers. It exhibits elevated expression in colorectal cancer tissues, contributing to the enhanced growth of SW480 and HCT116 colorectal cancer cells [41,42]. Additionally, *MALAT1* is deeply implicated in gastric carcinogenesis through diverse molecular pathways. For instance, it augments the proliferation of gastric cancer cells by downregulating the expression of miRNAs such as miR-122, miR-1297, miR-22-3p, and miR-202, and by repressing the activity of the oncogene *PCDH10*, thereby promoting the growth and invasiveness of gastric cancer [43].

Within the hub genes of subtype III, *SPARC* was shown to amplify the chemosensitivity of 5-FU by facilitating apoptosis. Our findings indicate that both cleaved *PARP* and cleaved caspase-3 levels were increased after overexpression of *SPARC* protein. Additionally, *Bax*, a pivotal protein in the apoptotic process, was significantly upregulated in SGC-7901 and BGC-823 cells with heightened *SPARC* expression. These outcomes implicate that *SPARC* may induce apoptosis in gastric cancer through the activation of the PARP/caspase-3 pathway [44]. Expression levels of the *SPARC* gene are notably correlated with clinical attributes of colorectal cancer, such as tumor stage, suggesting its potential as a biomarker for colorectal cancer [45].

### 3.3. Impact of Hub Genes on the Development of Gastrointestinal Tumors

Gene Ontology (GO), established by the Gene Ontology Consortium, serves as a comprehensive database that catalogs the functional roles of genes and their transcriptional and translational products within biological processes. Our analysis of the pathways involving hub genes in gastrointestinal cancer subtypes aims to delineate their biological functions and the pathways in which they participate.

**Figure 7:**
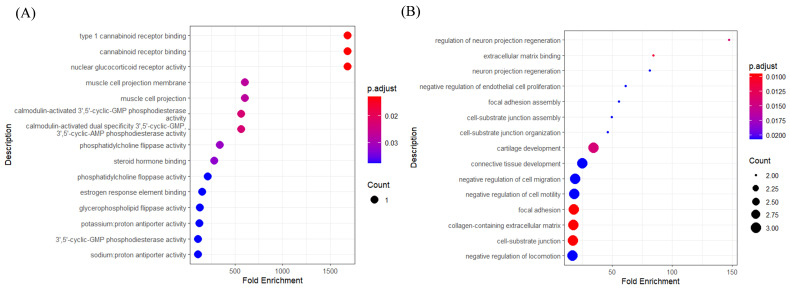
GO analysis results: (**A**) subtype I (**B**) subtype III.

Figure 7A illustrates the outcomes of GO analysis for subtype I with a primary focus on the Molecular Function (MF) category of GO. In this representation, dots correspond to distinct biological processes, and the magnitude of each dot is proportional to the gene count associated with the process. In addition, the color gradient reflects the adjusted *p*-value (p.adjust), denoting the statistical significance of enrichment. Cannabinoid receptors contribute to various intestinal physiological processes, including peristalsis, secretion, and epithelial barrier function. Research indicates that the deletion of cannabinoid receptor 1 can result in intestinal inflammation and cancer [46]. Activated cannabinoid receptors, notably CB1 and CB2, are recognized for their role in modulating inflammatory responses and tumor cell proliferation [47]. Glucocorticoid receptors are pivotal in regulating immune responses, inflammation, and cell survival. In the context of gastrointestinal cancers, glucocorticoids may modulate the tumor microenvironment via their receptors, potentially impacting tumor growth and metastasis by suppressing inflammation and regulating immune cell activity. Specifically, in colorectal cancer, glucocorticoids might facilitate cancer cell proliferation and invasion through the GR-CDK1 signaling pathway [48].

Figure 7B shows the results of GO analysis for subtype III, focusing mainly on the GO category “Biological Process (BP)”. Most of these functions are associated with extracellular matrix (ECM) interactions, cell migration, and tissue development. Engagement with the extracellular matrix is an essential component of the tumor microenvironment in gastrointestinal cancers, influencing the invasive and migratory capacities of tumor cells. The interaction between tumor cells and the ECM has the potential to either advance or retard tumor progression [49].

Genes like *MAGI2*, which encodes long non-coding RNA, and *MAGI2-AS3* and *RBMS3*, which encode the RNA Binding Motif Single Stranded Interacting protein3, participate in a spectrum of regulatory processes, encompassing signal transduction, gene-expression modulation, and intercellular communication. This participation may indicate that subtype I is particularly dynamic in the realms of cellular signaling and gene-expression regulation. Genes within subtype II might be more engaged in specialized regulatory roles, such as the involvement of non-coding RNA in transcriptional regulation, and could be pivotal in specific physiological or pathological contexts, including the modulation of gene expression in response to environmental stresses or disease conditions. Genes implicated in extracellular matrix interactions and tissue remodeling, such as *SPARC* and *FAP*, frequently contribute to tissue development, repair, and the cancer microenvironment. The roles of these genes suggest that subtype III may be instrumental in governing extracellular matrix dynamics and adaptations under pathological conditions.

## 4. Discussion

The prevalence of gastrointestinal cancers is escalating, most notably among younger demographics, constituting a substantial segment of malignant neoplasms within the digestive tract. The emergence of multiomics has unveiled the profuse heterogeneity of these tumors, a key determinant in their evolution, therapeutic response, and metastatic propensity. The intrinsic heterogeneity of GI tumors is reflected not only in differences in gene expression, but also in multi-level variations in the molecular, epigenetic, and immune microenvironment. These heterogeneities are crucial in explaining the differential performance of different tumor subtypes in terms of treatment response and prognosis. For instance, transcriptomic studies have revealed the intrinsic heterogeneity of tumor cells in gastrointestinal malignancies, highlighting the different molecular subtypes present in these tumors [50]. Furthermore, this heterogeneity is further influenced by tumor cell-intrinsic factors that lead to variability in immune cell infiltration and response to immunotherapy [51]. This heterogeneity creates challenges in clinical management of patients and affects treatment outcomes. Our study harnesses multimodal data analysis, integrating diverse technical approaches and data modalities, to deepen our understanding of the intrinsic tumor architecture, molecular makeup, and biological activities.

In our methodology, we integrate edge features derived from the optimized Canny operator in detecting a wide range of edges in images, along with transcriptomic and immunological data. Specifically, the significantly differential hsa-miR-21-5p between patient and normal samples has also been shown to regulate targets such as *TGFBI* and *PDCD4*, which were also found to be downregulated in our study. To forge a sample-similarity network, we conduct preliminary clustering on disparate data modalities to amplify the significance of localized similarities. This involves an iterative optimization of the similarity and kernel matrices, culminating in convergence. We then deploy spectral clustering on this integrated network to delineate distinct tumor subtypes.

The fusion of diverse data offers a comprehensive view of gene-expression profiles while also yielding insights into cellular and tissue architectures. This dual methodology highlights the tumor’s genotypic and phenotypic traits, leads to a comprehensive elaboration of distinct tumor subtypes, and hence, establishes a foundation for the development of therapeutic modalities.

We conducted an analysis of mRNA gene-expression profiles utilizing Weighted Gene Co-expression Network Analysis (WGCNA), identifying distinct gene modules. Synthesizing these findings with the outcomes of sample subtyping, we pinpointed the associated hub genes. These genes constitute critical regulatory pathways, the dysregulation or aberrant expression of which can significantly advance disease progression. Moreover, they represent promising therapeutic targets with the potential to modulate diverse network and pathway activities.

Our study demonstrates the profound utility of multimodal data analysis in elucidating the heterogeneity of gastrointestinal cancers, with significant implications for personalized medicine. By identifying different tumor subtypes, we can gain a better understanding of the unique molecular characteristics of each subtype. These features can be used clinically as diagnostic markers or therapeutic targets to aid in the development of individualized treatment plans. Key genes identified in different subtypes that are associated with tumor aggressiveness and metastasis may be potential candidates for future targeted therapies. By analyzing immune cell data, we can determine which patients are more likely to benefit from immunotherapy, enabling more targeted treatment selection in the clinic. For instance, in terms of immune cell composition, for subtype I, immune activators may enhance the immune response. For subtype II, anti-inflammatory or immunosuppressive drugs may be considered to reduce excessive immune responses. For subtype III, the focus is on promoting the homeostatic and restorative functions of the immune system.

While our optimized Canny operator has demonstrated remarkable efficacy in delineating tumor margin features, it might not encapsulate the full complexity of the tumor microenvironment. Therefore, while not within the scope of the present paper, we plan to pursue more sophisticated image-processing methodologies, including deep learning algorithms, with the aim of strengthening the detection and profiling of biomarkers within histopathological assessments.

## 5. Conclusions

Our study underscores the profound utility of multimodal data analysis in the study of gastrointestinal cancers and demonstrates that the integration of omics data can be achieved by seamlessly merging the edge features of tumor images with differential transcriptomic and immune cell data. The discovery of hub genes across various tumor subtypes paves the way for innovative diagnostic methods and tailored therapeutic strategies. Moreover, the integration multimodal data deepens insights into the intrinsic heterogeneity of gastrointestinal tumors. Overall, our results lay a robust groundwork for further investigating the complexities of GI cancers with the promise of advancing personalized medicine to achieve superior patient outcomes.

## Figures and Tables

**Table 1:** Hub genes for the three subtypes (sorted according to kWithin).

Subtype I	kWithin	Subtype II	kWithin	Subtype III	kWithin
MAGI2-AS3	371.7129	RP11-416A17.6	55.3853	SPARC	34.4698
TTC28	345.3234	RP11-166B2.3	52.2037	FAP	33.0935
RBMS3	345.2273	RP11-192H23.7	50.8855	BGN	29.6813
CNRIP1	338.6266	MALAT1	46.6874	SULF1	29.6049
PLEKHO1	323.7333	RP11-49O14.2	46.2525	CDH11	28.0017
GYPC	315.0139	CTD-2014D20.1	46.0766	PRRX1	26.9047
C20orf194	313.7970	LA16c-431H6.6	45.3105	THY1	26.4728
CLIP4	312.4037	NPIPB5	40.0239	NOX4	25.9135
FOXN3	309.4977	RYKP1	39.8201		
ATP8B2	300.8144				
RP11-875O11.1	286.9392				
PDE1A	254.0221				
NR3C1	249.1351				
SLC9A9	248.7150				
NR2F2-AS1	245.9516				
RP11-730A19.9	226.7302				

## Data Availability

The datasets ANALYZED for this study can be found in the following GitHub repository: https://github.com/gutmicrobes/GI-multimodal.git (accessed on 1 September 2024).

## Supplementary Materials

The following supporting information can be downloaded at: https://www.mdpi.com/article/10.3390/genes15091207/s1. Figure S1: Differences between cancer and normal samples; Figure S2: Reference chart for selecting soft thresholds for WGCNA analysis. Figure S3: Iterative process for four types of data; Table S1: Differential mRNA in tumor and normal samples. Table S2: Differential miRNA in tumor and normal samples. Table S3: Modules obtained from WGCNA analysis and the number of genes they contain.
