# Supplementary material for: Tumor Heterogeneity in Gastrointestinal Cancer Based on Multimodal Data Analysis
Source: Genes (Basel). 2024 Sep 13;15(9):1207. doi: 10.3390/genes15091207 (PMC11430818; doi:10.3390/genes15091207)
Supplement: Supplementary file 1 [file genes-15-01207-s001.zip › genes-3179686-supplementary.pdf]

Supplementary Material

1 Supplementary Methods

1.1 The bilateral filtering formulas

$$g(i,j) = \frac{\sum_{(m,n)\in S(i,j)} f(m,n) * w(i,j,m,n)}{\sum_{(m,n)\in S(i,j)} w(i,j,m,n)} \tag{1}$$

$$w(i,j,m,n) = d(i,j,m,n) * r(i,j,m,n) \tag{2}$$

$$d(i,j,m,n) = exp\left(-\frac{(i-m)^2 + (j-n)^2}{2 * \sigma_d^2}\right) \tag{3}$$

$$(i,j,m,n) = exp\left(-\frac{\parallel f(i,j) - f(m,n) \parallel^2}{2 * \sigma_r^2}\right). \tag{4}$$

In Equation (1), $g(i,j)$represents the output point, where $S(i,j)$ denotes the neighborhood range of size $(2 \times N + 1) \times (2 \times N + 1)$ centered at point (i,j), and $f(m,n)$ corresponds to the grayscale value of the pixel located at the matrix coordinates (m,n).

1.2 Calculation of the direction of pixel points

$$G = \sqrt{G_x^2 + G_y^2}, \qquad \theta = arctan\left(\frac{G_y}{G_x}\right). \tag{5}$$

We defined $\boldsymbol{G_x} = \boldsymbol{S_x} * \boldsymbol{I}$ and $\boldsymbol{G_y} = \boldsymbol{S_y} * \boldsymbol{I}$, where I denoted a 3x3 matrix of gray values centered on the pixel of interest. The Sobel kernels for capturing horizontal and vertical features were constructed as follows:

$$S_x = \begin{bmatrix} -1 & 0 & 1 \\ -2 & 0 & 2 \\ -1 & 0 & 1 \end{bmatrix} \qquad S_y = \begin{bmatrix} -1 & -2 & -1 \\ 0 & 0 & 0 \\ 1 & 2 & 1 \end{bmatrix}.$$

1.3 The equations for determining TH and TL

$$TL = 0.1 * max(H^*) \tag{6}$$

$$TH = 0.5 * max(H^*). \tag{7}$$

where H^*denoted the pixel matrix post nonmaximum suppression:

1.4 Nonlinear mapping of correlation coefficient matrix elements

$$a_{ij} = |a_{ij}|^\beta. \tag{8}$$

1.5 The multimodal data fusion process

1.5.1 Calculation of the TOM matrix

$$w_{\mathrm{ij}} = \frac{I_{ij} + a_{ij}}{min(k_i, k_i) + 1 - a_{ij}}. \tag{9}$$

the association between samples i and j was represented by $I_{ij} = \sum_{\mu} a_{iu} * a_{uj}$, and the association of sample i with the remaining samples was given by $k_i = \sum_{\mu} a_{iu}, \mu \neq i$.

1.5.2 Calculation of similarity matrix and kernel matrix

$$q_{ij} = exp\left(-\frac{\mathrm{d}_{\mathrm{ij}}^2}{0.5 * \varepsilon_{ij}}\right) \tag{10}$$

$$\varepsilon_{\mathrm{ij}} = \frac{\mathrm{mean}(\mathrm{d_i}, \mathrm{N_i}) + \mathrm{mean}(\mathrm{d_j}, \mathrm{N_j}) + \mathrm{d_{ij}}}{3}. \tag{11}$$

The distance matrix for the samples was calculated using Equations 10 and 11. The value $\mathrm{mean}(\mathrm{d_i}, \mathrm{N_i})$ represented the mean distance of sample i from all other samples, excluding itself.

A similarity matrix among samples was established, as detailed in equation (12):

$$P_{ij} = \begin{cases} \frac{q_{ij}}{2 * \sum_{k \neq i} q_{ik}}, i \neq j \\ \frac{1}{2}, i = j \end{cases}. \tag{12}$$

Utilizing the preclustered data, we formulated the kernel matrix as presented in Equation (13):

$$S_{ij} = \begin{cases} \frac{q_{ij}}{\sum_{k \in C_i} q_{ik}}, j \in C_i \\ 0, otherwise \end{cases}. \tag{13}$$

where C_i denoted the cluster assignment of sample i within the preclustered data categories, such as mRNA.

1.5.3 The Construction of the Fusion Matrix

We iteratively refined P_v for each data modality v={mRNA, miRNA, image data, immune cell data} using Equation (14) and quantified the iterative changes in the data in terms of Frobenius norms, as described by Equation (15):

$$M^v = S^v \times \frac{\sum_{k \neq v} P^k}{3} \times (S^v)^T \tag{14}$$

$$\| A \|_F = \sqrt{\sum_{i=1}^{m} \sum_{j=1}^{m} |a_{ij}|^2}. \tag{15}$$

2 Supplementary Figures and Tables

2.1 Supplementary Figures

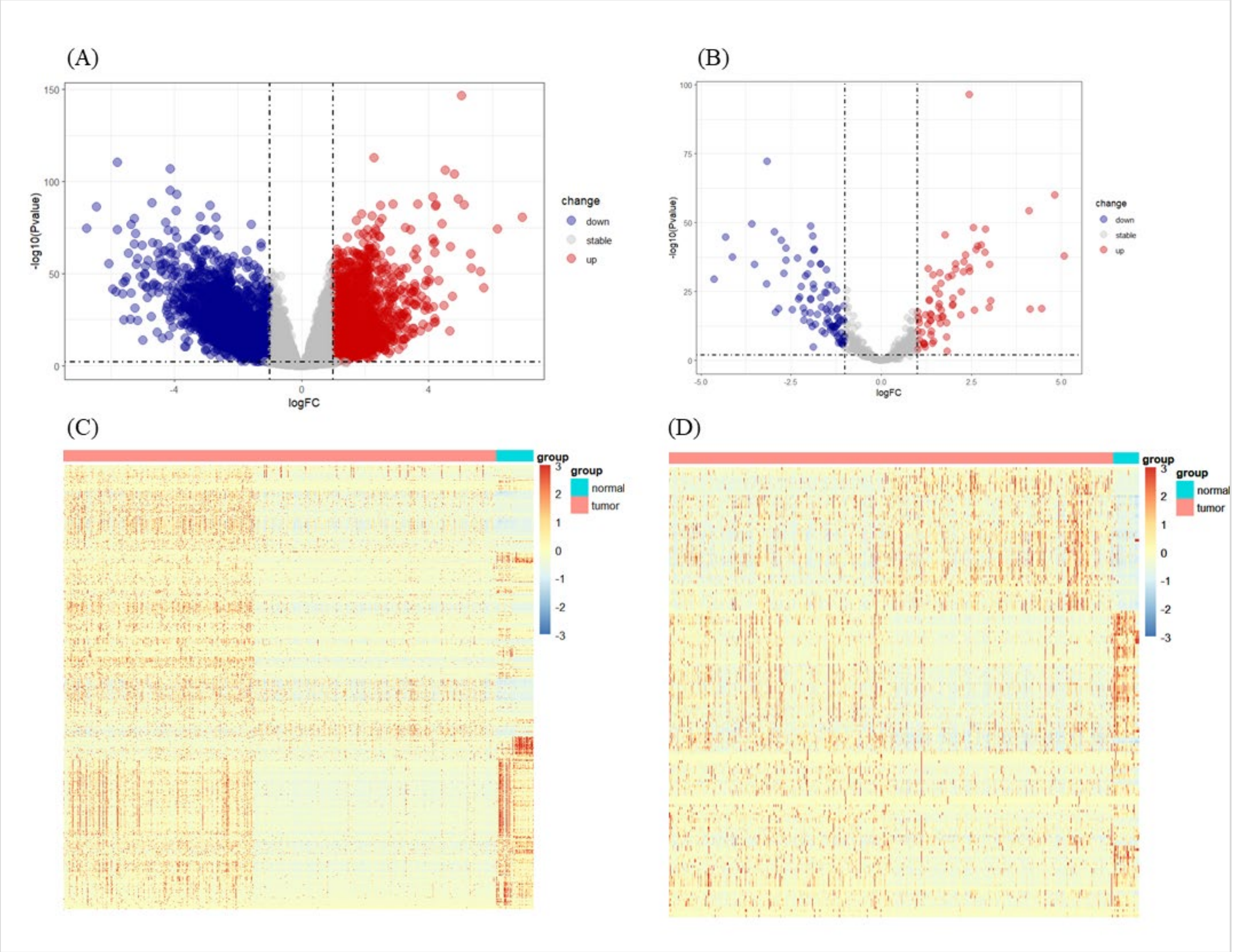

Figure S1. Differences between cancer and normal samples. **(A)** differential mRNA screening and **(B)** differential miRNA screening. **(C)** mRNA heat-map of cancer and normal samples; **(D)** miRNA heat map of cancer and normal samples.

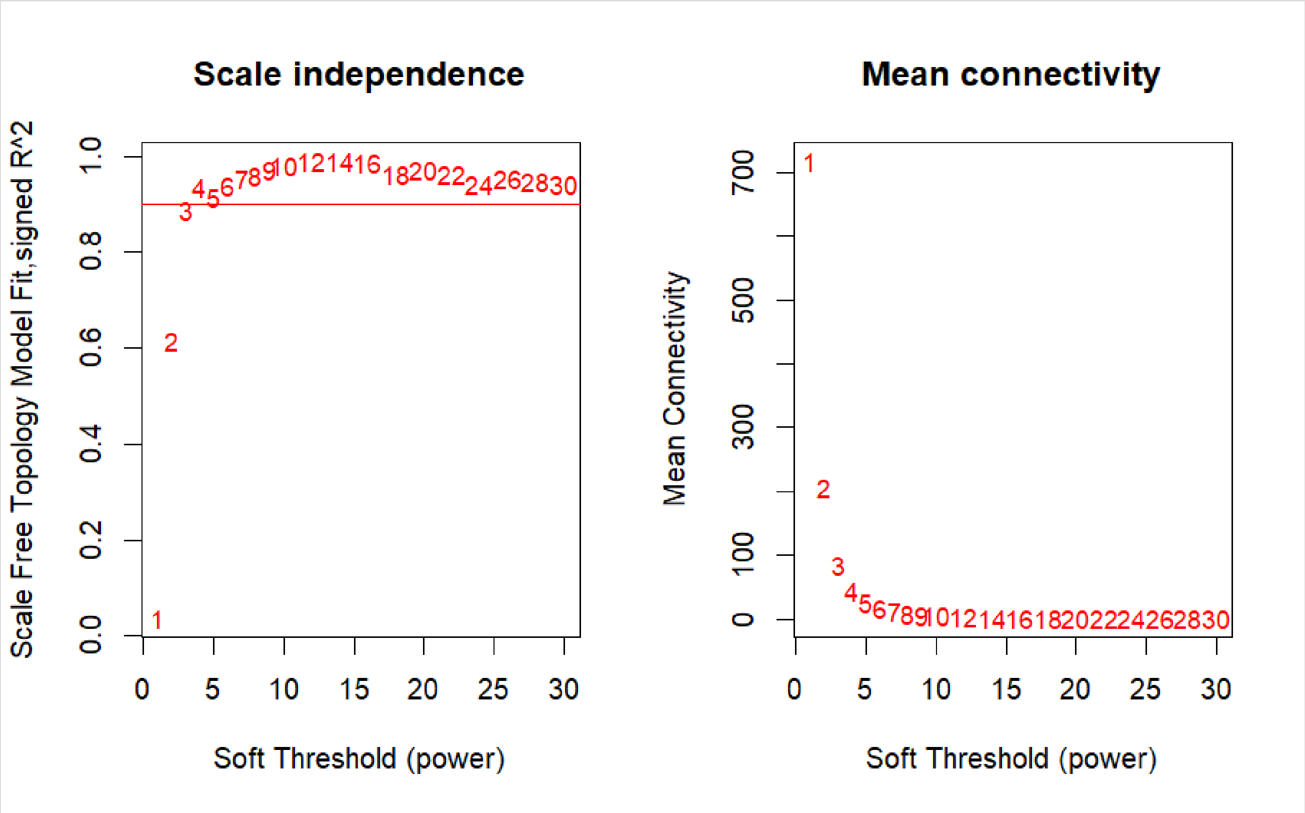

Figure S2. Reference chart for selecting soft thresholds for WGCNA analysis.

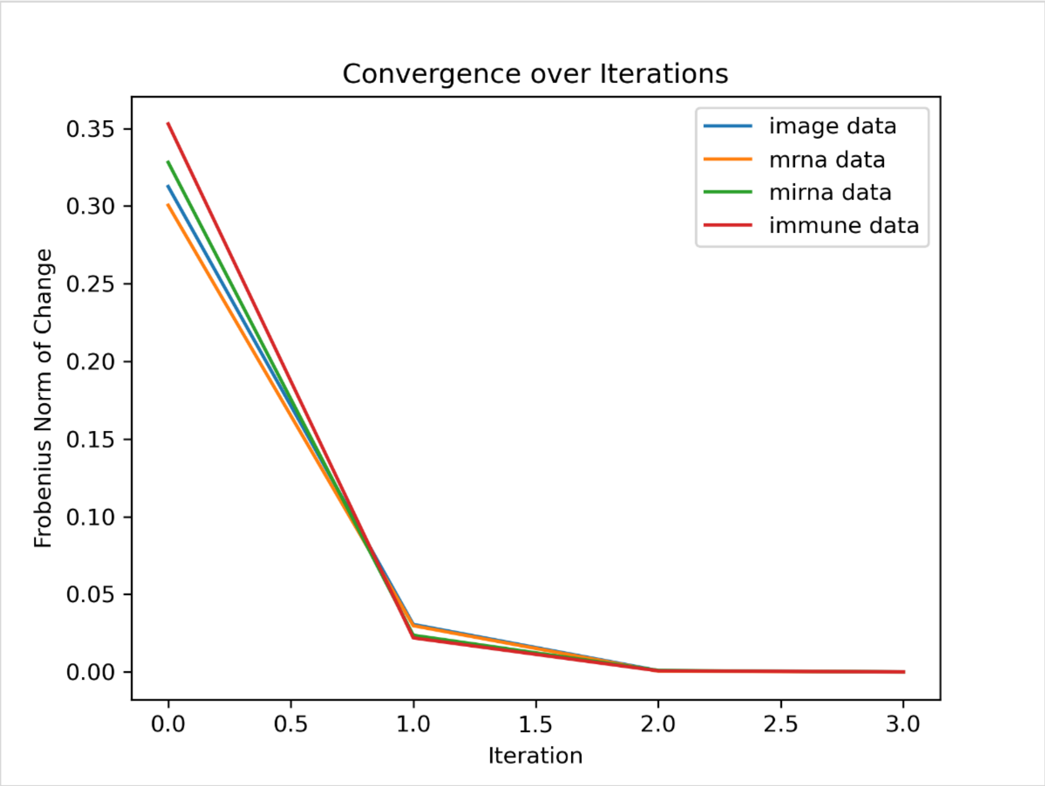

Figure S3. Iterative process for four types of data

2.2 Supplementary Tables

Table S1. Differential mRNA in tumor and normal samples

miRNA	logFC	P.Value	change	miRNA	logFC	P.Value	change
ESM1	5.0369	3.73E-147	up	LGI1	-4.6907	4.42E-89	down
MTHFD1L	2.2804	1.54E-113	up	ACAN	3.6641	1.71E-88	up
KRT24	-5.7884	6.26E-111	down	LRP8	2.8832	1.75E-88	up
CLEC3B	-4.1137	2.15E-107	down	KRT80	5.1263	5.59E-88	up
INHBA	4.5210	1.04E-106	up	ETV4	4.2458	7.30E-88	up
CDH3	4.8189	7.92E-105	up	MMP27	-2.8649	1.01E-87	down
CMTM5	-4.1173	1.05E-95	down	AJUBA	2.4903	1.45E-87	up
FAM180B	-3.9266	7.86E-94	down	GRIN2D	4.2187	2.44E-87	up
CLDN1	4.1461	2.05E-92	up	OTOP2	-6.4448	8.97E-87	down
WNT2	4.9464	4.72E-91	up	LYVE1	-3.9449	1.09E-84	down

Table S2. Differential miRNA in tumor and normal samples

miRNA	logFC	P.Value	change	miRNA	logFC	P.Value	change
hsa-miR-21-5p	2.4518	3.51E-97	up	hsa-miR-125a-5p	-1.8907	6.44E-46	down
hsa-miR-139-5p	-3.1549	6.45E-73	down	hsa-miR-133a-3p	-4.3151	1.78E-45	down
hsa-miR-196a-5p	4.8233	1.02E-60	up	hsa-miR-145-5p	-2.7684	2.93E-44	down
hsa-miR-135b-5p	4.1157	5.86E-55	up	hsa-miR-141-3p	2.7772	1.57E-42	up
hsa-miR-139-3p	-3.5757	3.66E-50	down	hsa-miR-183-5p	2.6797	2.73E-42	up
hsa-miR-378a-5p	-1.9494	2.21E-49	down	hsa-miR-195-3p	-2.6331	1.65E-41	down
hsa-miR-182-5p	2.5674	6.30E-49	up	hsa-miR-328-3p	-1.8465	4.99E-41	down
hsa-miR-141-5p	2.8951	2.33E-48	up	hsa-miR-194-5p	2.6350	7.50E-41	up

hsa-miR-486-5p	-2.9528	2.13E-47	down	hsa-miR-29b-2-5p	-1.8586	1.31E-40	down
hsa-miR-21-3p	1.7850	3.17E-46	up	hsa-miR-429	2.8898	8.12E-40	up

Table S3. Modules obtained from WGCNA analysis and the number of genes they contain.

**MODULE**	Black	Blue	Brown	Cyan	Green
**NUMBER**	168	1254	662	40	176
**MODULE**	Greenyellow	Yellow	Magenta	Midnightblue	Pink
**NUMBER**	78	202	90	33	163
**MODULE**	Purple	Red	Salmon	Tan	Turquoise
**NUMBER**	81	169	47	65	1747
